# The effect of etanercept on traditional metabolic risk factors for cardiovascular disease in patients with rheumatoid arthritis

**DOI:** 10.1007/s10067-016-3422-7

**Published:** 2016-10-05

**Authors:** Atul Deodhar, Bojena Bitman, Yue Yang, David H Collier

**Affiliations:** 1 Division of Arthritis and Rheumatic Diseases (OP09), Oregon Health and Science University, Portland, OR 97239 USA; 2 Amgen Inc., 1 Amgen Center Drive, Thousand Oaks, CA 91320 USA

**Keywords:** Cardiovascular disease, Rheumatoid arthritis, Tumor necrosis factor inhibitor

## Abstract

Patients with rheumatoid arthritis (RA) are at an increased risk of cardiovascular disease (CVD). Treatment with tumor necrosis factor inhibitors improves both joint symptoms associated with RA and also CVD risk. This exploratory analysis of a phase 4 study evaluated changes in metabolic risk factors in patients with RA treated with etanercept. Metabolic analytes were measured at baseline, week 12, and week 24 in patients enrolled in a randomized, double-blind, placebo-controlled study to evaluate the efficacy and safety of etanercept in moderately active RA. Patients received either placebo or etanercept 50 mg every week (QW) for 12 weeks, after which all patients received etanercept 50 mg QW through week 24. Levels of metabolic analytes were assessed in all patients, including patients with diabetes and hyperlipidemia, and described descriptively. A total of 210 patients were randomized, 104 to placebo and 106 to etanercept. There were no significant changes in metabolic risk factors from baseline to week 12 or 24 in all patients. Levels of metabolic analytes were similar in patients with diabetes and hyperlipidemia, with some exceptions; fasting glucose and fasting insulin decreased through week 12, and hemoglobin A1C decreased slightly through week 24 in patients with diabetes. Treatment with etanercept did not adversely affect levels of metabolic risk factors for CVD in patients with RA.

## Introduction

It is well documented that patients with rheumatoid arthritis (RA) are at an increased risk of cardiovascular disease (CVD) and experience higher rates of cardiovascular (CV) morbidity and mortality than the general population [1–4]. While traditional CVD risk factors—including cholesterol levels—may contribute to this heightened risk in patients with RA, they do not completely account for the increased incidence of CV events [5, 6]. In addition to traditional CVD risk factors, the systemic inflammation associated with RA also plays a role in increasing CVD risk in patients [7]. Decreasing inflammation is thought to decrease CV morbidity and mortality in patients with RA [8]. Tumor necrosis factor inhibitor (TNFi) therapy, in particular, has been shown to improve both RA symptoms and CVD risk and reduce CV morbidity and mortality [9, 10]. In this study, we measured the levels of selected metabolic analytes in patients with RA who received treatment with etanercept to further understand the effect of TNFi therapy on traditional CVD risk factors.

## Materials and methods

### Study design

The evaluation of CVD risk factors was an exploratory endpoint in a phase 4, prospective, randomized, double-blind, placebo-controlled study (NCT01313208) to evaluate the efficacy and safety of etanercept in patients with moderately active RA despite disease-modifying anti-rheumatic drug (DMARD) therapy. Patients were randomized 1:1 to receive 50 mg etanercept or placebo every week (QW) for 12 weeks, stratified by current methotrexate (MTX) use (yes/no). At week 12, all patients received 50 mg etanercept QW for another 12 weeks.

### Patients

Eligibility criteria have been previously published [11]. In particular, patients were required to have moderately active RA, as defined by their disease activity score based on 28 joints (DAS28) with C-reactive protein (CRP) as the indicator of inflammation (3.2 < DAS28-CRP ≤ 5.1).

### Subpopulations of interest

Patients were further categorized based on diabetes and hyperlipidemia. Patients with diabetes were defined as those with a current history of diabetes (stop date in medical history missing or after enrollment date), and/or taking insulin, and/or taking oral anti-diabetics. Patients with hyperlipidemia were defined as those with a current history of hyperlipidemia or hypercholesterolemia (stop date in medical history missing or after enrollment date) and/or taking statins.

### Objective

The objective of this study was to evaluate changes in metabolic CVD risk factors in patients with moderately active RA treated with etanercept.

### Outcomes

The metabolic analytes measured in this study were fasting glucose, fasting insulin, hemoglobin A1C, total cholesterol, high-density lipoprotein (HDL), low-density lipoprotein (LDL), triglycerides, apolipoprotein A1, apolipoprotein B, adiponectin, leptin, and plasma N-terminal pro b-type natriuretic peptide (NT-proBNP). Shifts in grade (low, normal, high) for each analyte from baseline to week 24 were also measured.

### Statistical analyses

Baseline demographics, disease characteristics, and metabolic parameters were summarized descriptively for the overall study population and diabetic and hyperlipidemic subpopulations by treatment groups and time points. Changes in metabolic parameters over time during the study are also displayed graphically.

### Human rights

The study protocol and informed consent form were approved by the applicable Independent Ethics Committee (IEC) or Institutional Review Board (IRB). Patients were informed of all aspects of the study, and the forms were obtained before patients entered the study.

## Results

### Demographic and clinical characteristics at baseline

**Fig. 1:**
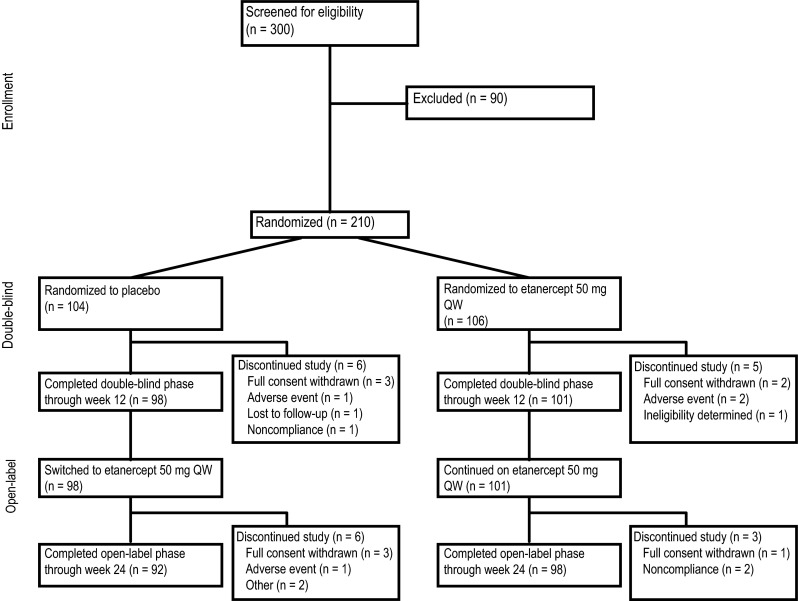
Patient disposition. The flow of patients from screening through week 24 of the study. QW every week

A total of 210 patients were enrolled in this study. During the first 12 weeks, 104 patients were randomized to placebo and 106 patients were randomized to etanercept (Table 1). The double-blind phase was completed by 95 % of patients: 94 % placebo and 95 % etanercept (Fig. 1). The open-label phase was completed by 91 % of patients: 89 % placebo-etanercept and 93 % etanercept-etanercept. Overall, the patients had a mean (standard deviation [SD]) age of 56 (12) years, were 77 % female, were 87 % white, had a mean (SD) RA duration of 8 (10) years, and had a mean (SD) disease severity (as measured by DAS28-CRP) of 4.9 (0.8) (Table 1). There were 14 % of patients with a medical history of diabetes and 30 % with a medical history of hyperlipidemia or hypercholesterolemia. There were 22 % of patients receiving statins, 11 % anti-diabetic oral agents, 3 % insulin, and 52 % prednisone.

**Table 1:** Demographic and clinical characteristics at baseline

Characteristic	Placebo-etanercept	Etanercept-etanercept	Total
	*n* = 104	*n* = 106	*n* = 210
Sex (female), *n* (%)	86 (82.7)	75 (70.8)	161 (76.7)
Race (white), *n* (%)	90 (86.5)	93 (87.7)	183 (87.1)
Age (years), mean (SD)	55.5 (12.8)	56.5 (12.1)	56.0 (12.4)
DAS28-CRP, mean (SD)	4.9 (0.8)	4.9 (0.7)	4.9 (0.8)
CRP (mg/L), mean (SD)	9.4 (16.3)	7.6 (11.8)	8.5 (14.2)
ESR (mm/h), mean (SD)	30.8 (23.5)	30.3 (22.8)	30.6 (23.1)
Tobacco, *n* (%)			
Never	49 (47.1)	54 (50.9)	103 (49.0)
Former	35 (33.7)	34 (32.1)	69 (32.9)
Current	20 (19.2)	18 (17.0)	38 (18.1)
RA duration (years), mean (SD)	7.4 (8.1)	8.3 (11.2)	7.8 (9.8)
RA medication history, *n* (%)	103 (99.0)	106 (100.0)	209 (99.5)
DMARDs (nonbiologic)	103 (99.0)	105 (99.1)	208 (99.0)
NSAIDs	70 (67.3)	75 (70.8)	145 (69.0)
Corticosteroids	68 (65.4)	73 (68.9)	141 (67.1)
Analgesics	44 (42.3)	38 (35.8)	82 (39.0)
Biologics	10 (9.6)	10 (9.4)	20 (9.5)
Other	1 (1.0)	0 (0.0)	1 (0.5)
Type 2 diabetes mellitus^a^, *n* (%)	12 (11.5)	17 (16.0)	29 (13.8)
Hyperlipidemia^b^, *n* (%)	30 (28.8)	33 (31.1)	63 (30.0)
Other medication history, *n* (%)			
Statin	21 (20.2)	26 (24.5)	47 (22.4)
Insulin	2 (1.9)	4 (3.8)	6 (2.9)
Oral anti-diabetic	10 (9.6)	12 (11.3)	22 (10.5)
Prednisone	53 (51.0)	57 (53.8)	110 (52.4)

*SD* standard deviation, *DAS28* disease activity score based on 28 joints, *CRP* C-reactive protein, *ESR* erythrocyte sedimentation rate, *RA* rheumatoid arthritis, *DMARD* disease-modifying anti-rheumatic drug, *NSAID* nonsteroidal anti-inflammatory drug

^a^Patients with type 2 diabetes mellitus were defined as those with a current history of diabetes (stop date in medical history missing or after enrollment date), and/or taking insulin, and/or taking oral anti-diabetics

^b^Patients with hyperlipidemia were defined as those with a current history of hyperlipidemia or hypercholesterolemia (stop date in medical history missing or after enrollment date) and/or receiving statins

### Changes in metabolic analytes

Levels of CRP decreased in the overall study population by week 24, but there were no significant changes in any other metabolic analytes measured from baseline to weeks 12 and 24 in all patients (Table 2). There were rare elevations in liver function tests and none greater than three times normal.

**Table 2:** Summary of analytes in all patients, patients with diabetes, and patients with hyperlipidemia

Analyte, mean (SD)	Visit	All		Diabetes		Hyperlipidemia	
		PBO-ETN	ETN-ETN	PBO-ETN	ETN-ETN	PBO-ETN	ETN-ETN
		*n* = 104	*n* = 106	*n* = 12	*n* = 17	*n* = 30	*n* = 33
Fasting glucose, mg/dL	Baseline	99.0 (30.7)	98.6 (30.3)	143.0 (69.2)	142.3 (53.1)	113.8 (46.2)	111.4 (43.5)
	Week 12	95.9 (22.3)	97.3 (26.6)	127.3 (42.4)	133.1 (47.1)	104.3 (32.1)	105.9 (29.3)
	Week 24	101.0 (22.8)	100.1 (24.0)	134.8 (35.9)	138.4 (35.8)	105.3 (26.0)	107.6 (17.3)
Fasting insulin, mIU/L	Baseline	14.3 (17.6)	16.0 (34.9)	24.4 (18.3)	40.3 (79.5)	22.4 (28.5)	26.8 (60.6)
	Week 12	15.3 (22.4)	13.2 (13.5)	20.2 (15.0)	16.1 (21.5)	18.0 (16.2)	17.7 (17.3)
	Week 24	15.0 (18.4)	12.0 (12.1)	23.0 (22.7)	14.8 (14.0)	14.6 (11.9)	14.4 (12.8)
Hemoglobin A1C, %	Baseline	5.7 (0.9)	5.7 (0.7)	7.2 (1.9)	6.7 (1.2)	6.1 (1.4)	6.1 (1.0)
	Week 12	5.7 (0.9)	5.6 (0.6)	7.0 (2.1)	6.7 (0.8)	5.9 (1.3)	6.0 (0.8)
	Week 24	5.6 (0.9)	5.6 (0.6)	6.8 (1.9)	6.5 (0.8)	5.9 (1.2)	5.8 (0.6)
Total cholesterol, mg/dL	Baseline	195.2 (44.2)	186.6 (37.1)	204.0 (32.9)	171.9 (36.6)	205.1 (55.1)	181.6 (42.5)
	Week 12	190.9 (40.7)	184.8 (37.4)	205.6 (36.2)	170.0 (33.7)	190.7 (44.2)	182.6 (44.5)
	Week 24	197.0 (41.7)	191.1 (39.6)	185.5 (32.4)	179.9 (37.3)	192.8 (44.4)	190.5 (43.2)
HDL, mg/dL	Baseline	62.0 (18.1)	62.1 (21.7)	52.6 (13.2)	57.8 (17.1)	57.4 (12.3)	58.5 (18.0)
	Week 12	60.5 (17.1)	61.6 (18.3)	54.7 (13.6)	61.9 (19.5)	56.9 (14.0)	61.4 (19.1)
	Week 24	62.1 (18.5)	62.2 (22.9)	52.1 (14.5)	60.6 (15.5)	56.0 (13.8)	62.1 (18.4)
LDL, mg/dL	Baseline	105.6 (36.3)	97.8 (30.0)	118.4 (26.9)	81.8 (29.5)	109.0 (40.7)	92.7 (37.2)
	Week 12	103.2 (35.4)	96.9 (33.2)	112.1 (32.3)	79.3 (31.5)	102.2 (38.2)	90.3 (39.3)
	Week 24	105.5 (35.4)	101.7 (31.0)	101.9 (89.1)	89.1 (31.5)	99.0 (34.3)	100.0 (33.3)
Triglycerides, mg/dL	Baseline	135.2 (91.6)	133.1 (80.1)	164.7 (68.6)	163.2 (91.6)	183.8 (134.1)	154.2 (78.0)
	Week 12	139.2 (85.5)	131.8 (69.4)	221.9 (170.3)	153.8 (71.8)	167.2 (113.4)	155.0 (67.8)
	Week 24	149.6 (104.0)	136.6 (76.2)	192.8 (165.2)	150.7 (71.1)	189.8 (141.0)	142.0 (74.9)
Apolipoprotein A1, mg/dL	Baseline	159.6 (27.8)	160.5 (35.4)	152.6 (23.2)	158.5 (32.7)	158.9 (26.2)	159.7 (32.8)
	Week 12	159.2 (28.1)	162.0 (32.8)	154.8 (25.3)	164.1 (30.3)	157.5 (27.9)	167.8 (34.2)
	Week 24	163.7 (32.1)	161.6 (38.0)	150.2 (25.7)	163.5 (22.5)	161.3 (29.8)	167.1 (33.4)
Apolipoprotein B, mg/dL	Baseline	91.6 (25.5)	86.2 (22.2)	106.4 (24.1)	79.7 (22.3)	101.7 (28.4)	86.3 (24.5)
	Week 12	89.5 (24.7)	84.9 (23.8)	107.3 (25.6)	79.6 (20.7)	95.5 (26.3)	85.8 (25.9)
	Week 24	91.8 (24.2)	88.7 (25.0)	91.9 (25.3)	83.5 (22.9)	95.4 (25.2)	91.4 (26.9)
Adiponectin, mg/L	Baseline	11.6 (6.8)	11.5 (8.7)	6.8 (3.4)	7.8 (3.8)	9.2 (4.7)	10.5 (8.2)
	Week 12	11.7 (7.4)	11.6 (8.8)	7.3 (3.3)	8.4 (4.4)	9.0 (4.4)	10.9 (9.0)
	Week 24	12.3 (7.2)	11.6 (9.1)	7.7 (3.7)	8.1 (4.2)	9.7 (4.6)	11.5 (9.6)
Leptin, μg/L	Baseline	29.3 (20.9)	33.5 (28.5)	31.5 (23.0)	42.8 (36.6)	33.1 (17.0)	41.5 (30.2)
	Week 12	30.1 (22.7)	35.6 (30.9)	30.9 (29.8)	44.1 (31.6)	34.3 (18.8)	43.5 (32.1)
	Week 24	30.8 (22.7)	34.5 (28.9)	33.7 (25.7)	39.3 (25.5)	34.1 (22.6)	37.8 (22.8)
Plasma NT-ProBNP, ng/L	Baseline	242.0 (554.1)	170.1 (334.6)	242.1 (513.2)	151.0 (153.8)	484.0 (935.9)	210.8 (535.4)
	Week 12	232.2 (630.8)	168.2 (297.8)	236.8 (465.8)	114.4 (126.5)	482.8 (1116.5)	158.3 (266.7)
	Week 24	244.2 (687.5)	164.2 (315.7)	199.8 (382.8)	157.5 (229.6)	522.4 (1212.0)	174.3 (351.1)
CRP, mg/L	Baseline	9.4 (16.3)	7.6 (11.8)				
	Week 12	8.6 (10.8)	4.2 (6.2)				
	Week 24	6.2 (14.0)	4.3 (5.5)				

*SD* standard deviation, *PBO* placebo, *ETN* etanercept, *IU* international unit, *HDL* high-density lipoprotein, *LDL* low-density lipoprotein, *NT-ProBNP* N-terminal pro b-type natriuretic peptide, *CRP* C-reactive protein

**Fig. 2:**
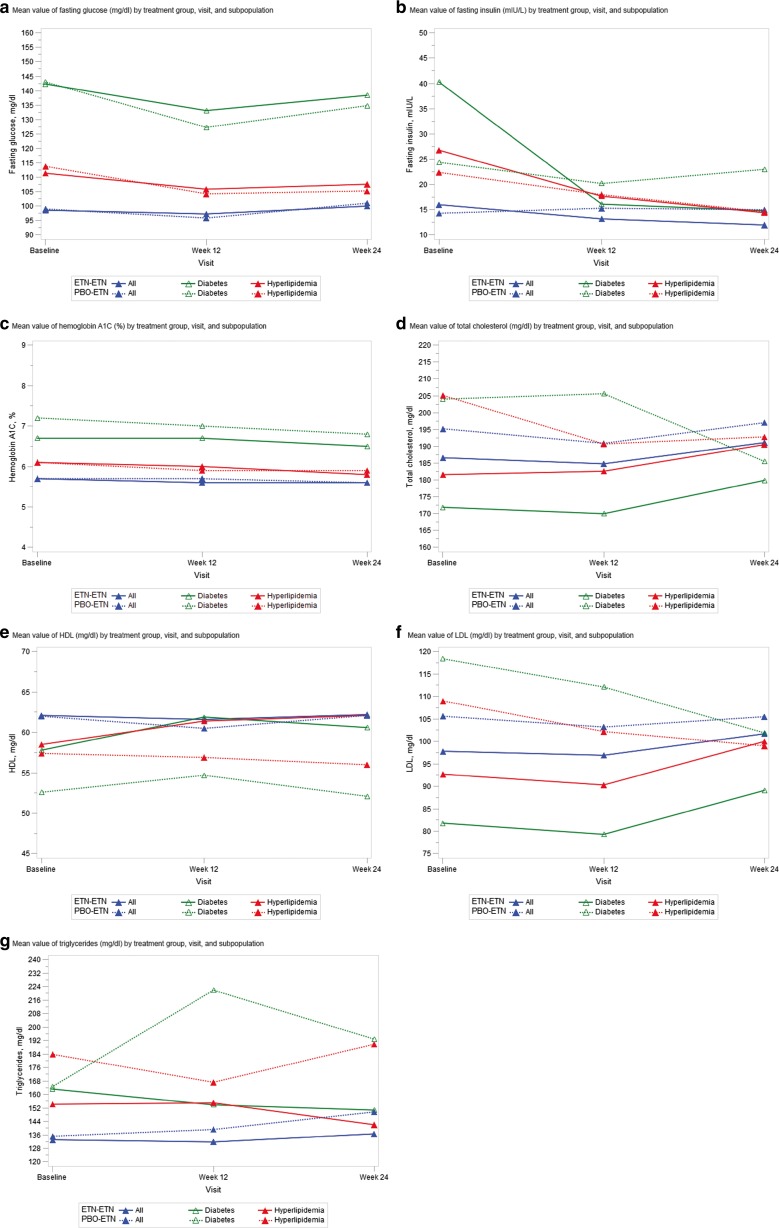
Changes in metabolic analytes from baseline to weeks 12 and 24. Changes in levels of **a** fasting glucose (mg/dL), **b** fasting insulin (mIU/L), **c** hemoglobin A1C (%), **d** total cholesterol (mg/dL), **e** HDL (mg/dL), **f** LDL (mg/dL), and **g** triglycerides (mg/dL) in all patients (*blue line*); patients with diabetes (*green line*); and patients with hyperlipidemia (*red line*) who received placebo/etanercept (*dotted line*) or etanercept/etanercept (*solid line*) from baseline to weeks 12 and 24. *HDL* high-density lipoprotein, *LDL* low-density lipoprotein

The metabolic analytes in patients with diabetes and hyperlipidemia did not change substantially from baseline to week 12 or 24 and were similar to those in the total study population, with some exceptions (Table 2 and Fig. 2). In patients with diabetes, fasting glucose decreased, HDL increased, and LDL decreased through week 12 in those receiving etanercept. In both subpopulations, fasting insulin decreased through week 12 and hemoglobin A1C decreased slightly through week 24. In addition, apolipoprotein A1 increased (with the exception of patients with diabetes receiving placebo-etanercept) and adiponectin increased slightly through week 24; leptin and apolipoprotein B did not change. Patients at a higher CVD risk experienced no or minimal changes in metabolic analytes from baseline to weeks 12 and 24. All changes that patients did experience were neither statistically nor clinically significant. Metabolic analytes were also compared between patients receiving concurrent nonsteroidal anti-inflammatory drugs and those who were not, among the total study population, patients with diabetes, and patients with hyperlipidemia; responses were similar between groups (data not shown).

### Shifts in grade for analytes

For the majority of patients, all metabolic analytes were in the normal range at baseline. The majority of patients completed the study week 24 at a normal grade for each analyte measured. Fewer than 25 % of patients in either treatment group experienced a shift in grade between baseline and week 24 for each metabolic analyte measured. Among those who did experience a shift in grade, patients were fairly evenly divided between those who improved in grade and those who worsened.

## Discussion

Treatment with etanercept did not adversely or positively affect the levels of traditional metabolic CVD risk factors in patients with RA. There were no significant changes in these metabolic analytes despite improvements in RA activity parameters, as indicated by decreases in CRP in both groups on etanercept by week 24. Previously reported improvements in CVD risk associated with TNFi therapy may stem from changes in other factors such as decrease in systemic inflammation. Results seen in patients with diabetes and hyperlipidemia demonstrated that there were no changes in traditional metabolic CVD risk factors, even in patients who were at a heightened risk of CVD.

Patients with RA exhibit a lipid paradox, in which patients with untreated RA have lower total cholesterol, HDL, and LDL than the general population, in which these lipid parameters are conventionally at elevated levels when patients are at a higher risk for CVD [5, 12, 13]. A potential explanation is that higher levels of CRP have been shown to decrease levels of lipids [6]. Elevated levels of CRP have been shown to independently correlate with increased CVD risk; a previous study has shown that elevated CRP was associated with an up to threefold increase in the risk of a heart attack [14]. Treatment to control RA subsequently results in an increase in total cholesterol, HDL, and LDL, without a concomitant increase in CVD risk; and in some cases even a decrease in CVD risk [6, 12, 13, 15, 16]. In our study, patients did not experience any change in levels of lipids, similar to some previous studies [15, 17].

Treatment with TNFi therapy, including etanercept, has been shown to lower CVD risk in patients with RA [18–20]. Based on analyses in this study and others, the reduction in CVD risk in patients with RA cannot be fully explained by changes in traditional metabolic risk factors. Managing RA symptoms and reducing inflammation in these patients appears to play a key role to decreasing CVD risk [8]. One study has even shown that the degree to which TNFi therapy reduces disease activity is proportional to the reduction in CVD risk [19]. One possible explanation behind the beneficial effect of TNFi therapy on CVD risk may relate a reduction in overall inflammation to a reduction in aortic inflammation. Findings from Maki-Petaja and colleagues demonstrated that TNFi therapy reduced aortic inflammation in patients with RA after 8 weeks of treatment, and this effect correlated with a decrease in aortic stiffness [21]. This study provides further evidence that the main effect of TNFi therapy is on inflammation, which can directly decrease inflammation in the aorta, preventing plaque formation or progression. Similar to reports on TNFi therapy, other biologics (e.g., interleukin-6 receptor inhibitor) also worsen the lipid profile with no worsening of CVD risk [22, 23].

The well-established benefit of TNFi therapy on CVD risk in patients with RA may also not be apparent in the short term. Recent work by Charles-Schoeman and colleagues demonstrated that patients with early RA in the treatment of early aggressive rheumatoid arthritis (TEAR) trial experienced increases in lipids (total cholesterol, LDL, and HDL) through 24 weeks of treatment with etanercept; these levels began to decrease by 48 weeks of treatment, with statistically significant reductions by week 102 compared with week 24 [24]. Given this timeline, the time periods evaluated in this manuscript—12 and 24 weeks—may not have been long enough to capture the long-term effects of etanercept treatment on metabolic risk factors. In addition, the study reported here recruited patients with moderate RA with mean a DAS28 of 4.9, compared with the patients with early RA in the TEAR trial who had mean DAS28 ranging from 5.48 to 5.82. The lack of high disease activity in this study may have had an effect on baseline lipid levels, and the small changes in disease activity in response to treatment with etanercept may have had a lesser impact on metabolic parameters. Another possibility why this study did not show any positive effect of etanercept on traditional CVD risk factors may be that this study was underpowered. Larger studies are required to confirm if this conclusion holds true in different populations.

In managing the CVD risk in patients with RA, it is important to evaluate both traditional CVD risk factors and RA severity. As shown in one study, incorporating both sets of factors into a model enhances its ability to predict the incidence rate of CV events [25]. Furthermore, as described in the European League Against Rheumatism (EULAR) recommendations for CVD risk management in patients with RA, the control of both systemic inflammation and traditional CVD risk factors is important to control CVD risk [26, 27]. The analysis presented here shows that etanercept controls the systemic inflammation in patients with RA, as evidenced by the reduction of CRP, and does not adversely affect levels of traditional cardiovascular risk factors.
